# Estimates of gene flow and dispersal in wild riverine Brook Trout (*Salvelinus fontinalis*) populations reveal ongoing migration and introgression from stocked fish

**DOI:** 10.1002/ece3.4556

**Published:** 2018-11-14

**Authors:** Spencer A. Bruce, Jeremy J. Wright

**Affiliations:** ^1^ Department of Biological Sciences University at Albany – State University of New York Albany New York; ^2^ New York State Museum Albany New York

**Keywords:** dispersal, gene flow, introgression, landscape genetics, migration, *Salvelinus fontinalis*

## Abstract

As anthropogenic impacts accelerate changes to landscapes across the globe, understanding how genetic population structure is influenced by habitat features and dispersal is key to preserving evolutionary potential at the species level. Furthermore, knowledge of these interactions is essential to identifying potential constraints on local adaptation and for the development of effective management strategies. We examined these issues in Brook Trout (*Salvelinus fontinalis*) populations residing in the Upper Hudson River watershed of New York State by investigating the spatial genetic structure of over 350 fish collected from 14 different sampling locations encompassing three river systems. Population genetic analyses of microsatellite data suggest that fish in the area exhibit varying degrees of introgression from nearby State‐directed supplementation activities. Levels of introgression in these populations correlate with water‐way distance to stocking sites, although genetic population structure at the level of individual tributaries as well as their larger, parent river systems is also detectable and is dictated by migration and influenced by habitat connectivity. These findings represent a significant contribution to the current literature surrounding Brook Trout migration and dispersal, especially as it relates to larger interconnected systems. This work also suggests that stocking activities may have far‐reaching consequences that are not directly limited to the immediate area where stocking occurs. The framework and data presented here may aid in the development of other local aquatic species‐focused conservation plans that incorporate molecular tools to answer complex questions regarding diversity mapping, and genetically important conservation units.

## 1 INTRODUCTION

A major goal of conservation biology is to determine how anthropogenic influences are shaping wild populations and their genetic structure under altered habitat regimes (Bushar et al., 2014; Cornille et al., 2015; Inoue, Lang, & Berg, 2015). Centuries of human mitigated translocations have led to intraspecific hybridization across the landscape, often leading to a reduction in genetic variability while increasing genetic homogenization (Laikre, Schwartz, Waples, & Ryman, 2010; Ozerov et al., 2016). These changes in genetic structure have the potential to negatively impact native populations by transforming a population's natural genetic constitution, which may be synonymous with local adaption, in the form of allele replacement and/or gene‐complex disruption (Edmands, 2007; Evans, Mekel‐Bobrov, Vallender, Hudson, & Lahn, 2006; Laikre et al., 2010). In addition to potentially negative genetic effects, the introduction of nonnative conspecifics produces risks associated with the introduction of pathogens, parasites, and disease (Adlard, Miller, & Smit, 2015; Cunningham, 1996; Gaughan, 2001). Understanding how the intentional transference of nonsympatric individuals is influencing wild populations is therefore key to executing successful management plans at the species level (Teixeira, Azevedo, Mendl, Cipreste, & Young, 2007). In addition to considerations related to translocations and introgressive hybridization, understanding how gene flow and migration patterns are affected by landscape features is also imperative to addressing concerns related to reproduction, dispersal, and population viability (Couvet, 2002; Young, Boyle, & Brown, 1996).

Habitat fragmentation, the division of habitat into smaller and more isolated patches divided by a matrix of unnatural barriers, has the potential to greatly reduce gene flow and connectivity between individuals or populations of the same species (Fahrig, 2003). In situations where populations have become small and selection pressures are heavy, gene flow from human mediated transference of conspecifics has the potential to increase population sizes even if the resulting phenotypes are unsuited to the environment, in turn leading to increased genetic variation which over time may potentially allow for new adaptations (Holt & Gomulkiewicz, 1997; Sexton, Hangartner, & Hoffmann, 2014). Given the complexities associated with translocations and habitat connectivity, understanding how landscape structure and genetic structure are linked is key to making decisions about proper management strategies under altered habitat regimes.

**Figure 1:**
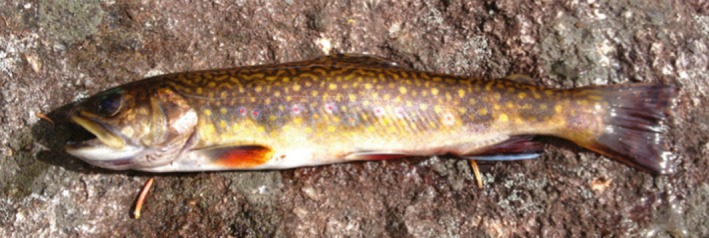
Brook Trout (*Salvelinus fontinalis)*

The Upper Hudson River watershed is located in the Northeastern United States, and is a major feeder of the Hudson River, which flows south through New York State, terminating at the tip of Manhattan in New York City where it meets the Atlantic Ocean. The Upper Hudson River watershed is located in the Adirondack Park, and is surrounded by dense protected wild forest, making large areas of the watershed inaccessible, and it is therefore one of the most pristine remote watersheds in the State. Among the many fish species found in the Upper Hudson River watershed, wild Brook Trout (*Salvelinus fontinalis*; Figure 1) are among the most likely to be negatively impacted by habitat alterations (Merriam, Fernandez, Petty, & Zegre, 2017). Currently, over 300 lakes and ponds in New York State are actively managed as Brook Trout habitat, with records of wild reproduction in over a hundred (Baker et al., 1990). Despite this fact, the number of wild, unstocked, self‐sustaining populations in New York is considered to be far lower, and is projected to make up only 5% of the total number of water bodies that have been sampled (Baker et al., 1990). In addition, New York was one of the first states in the United States to supplement wild populations for recreational fisheries enhancement. State‐based stocking of Brook Trout began in the late 1800s (Daniels, 2011; Emery, 1985), and New York State currently maintains six strains of Brook Trout for stocking on an annual basis (NYSDEC, 2017).

Given that New York Brook Trout have been manipulated with supplemental stocking, populations have been disconnected due to habitat fragmentation, and water quality declines have reduced viable habitat, sorting out present‐day genetic structure has become exceedingly complex (Perkins, Krueger, & May, 1993a, 1993b; Bruce, Hare, Mitchell, & Wright, 2018). Based on current climate projections, the United States Environmental Protection Agency (EPA) has predicted a 50%–100% decline in Brook Trout abundance for the region by the year 2100 (E.P.A., 2015). These predictions are compounded by myriad studies published over the past several decades that have elucidated the potential negative effects of stocking on top of native populations (Araki, Berejikian, Ford, & Blouin, 2008; Fraser, 2008; Laikre & Ryman, 1996; Laikre et al., 2010; Rhymer & Simberloff, 1996; Ryman & Laikre, 1991). The degree to which Brook Trout populations have been, and are predicted to be, impacted by direct and indirect anthropogenic factors makes it critical that regions exhibiting heightened levels of effective population size and comparatively distinct genetic structure be distinguished and preserved (Ficke, Myrick, & Hansen, 2007; Gao et al., 2012). Understanding these patterns of biogeographic structure is therefore essential to maintaining viability and evolutionary potential, not just for Brook Trout, but for all species across changing landscapes (Abdul‐Muneer, 2014; Bruce et al., 2018).

**Figure 2:**
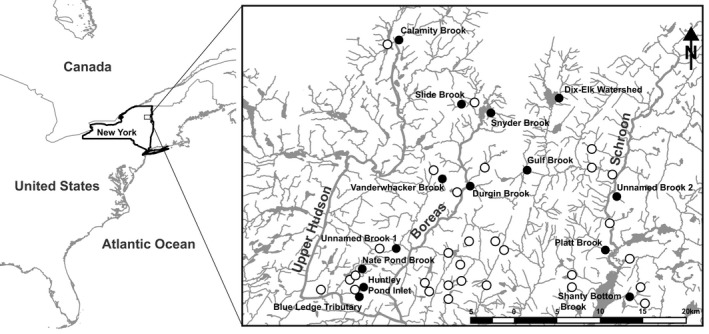
Map of North America, New York State demarcated in black with inset of study area. Labeled sample site locations represent the geographic center of sampling, while open circles demarcate proximal stocking locations. Hydrology is demarcated in gray while the major river systems are also labeled in gray

In the Upper Hudson River watershed, three main river systems act as the major hydrological feeders to the downstream Hudson River. These rivers include the Boreas River, the Schroon River, and the Upper Hudson itself (Figure 2). These river systems are substantially larger than their feeder tributaries, and provide habitat for a wide range of nonnative competitors and potential predators (Greeley, 1955; Loukmasa & Perryb, 2015). In addition, all three of these river systems experience summer high temperatures in the range of 20–25°C, making them a potential thermal stressor for wild Brook Trout and their offspring (SHEDS Development Team, 2017). Nevertheless, the river systems that comprise the Upper Hudson drainage are unique in that they are fed by a number of tributaries that possess ideal Brook Trout habitat (DeWeber & Wagner, 2015). The Upper Hudson drainage has, however, been subjected to decades of state‐sanctioned stocking activities in the main stem of all three of these systems (Van Offelen, Krueger, & Schofield, 1993; Webster & Flick, 1981). Despite the long‐term interest in, and attempted management of, this fishery, little is currently known about Brook Trout population genetic structure in this region (but see Bruce et al., 2018; Perkins et al., 1993a, 1993b) or the potential impacts, genetic or otherwise, of these stocking activities on naturally occurring Brook Trout populations.

In addition to considerations related to supplemental stocking, a number of recent studies have shown relatively short dispersal distances for Brook Trout, suggesting that the median stream channel distances between pairs of individuals belonging to the same families are as little as 100–250 m, with significant correlations between the locations of parents and their offspring (Hudy, Coombs, Nislow, & Letcher, 2010; Kanno, Vokoun, & Letcher, 2011). Nevertheless, little information is available on rates and magnitude of dispersal and/or gene flow between Brook Trout populations that are potentially connected by such larger riverine systems. If nonnative competitors, habitat fragmentation or water quality issues in this watershed dictate Brook Trout migration, we would expect strong genetic population structure based on limited gene flow between feeder tributaries, with a strong signal of isolation by distance. We would also expect minimal signs of introgression from stocked fish, comparatively low measures of genetic diversity and effective population size, and limited evidence of migration between sampling sites. On the other hand, if Brook Trout migration in the Upper Hudson watershed is widespread, with high summer temperatures, landscape features, and nonnative competitors having little influence on dispersal and gene flow in the cooler months, we would expect genetic population structure to be less pronounced. Patterns under this scenario would include strong admixture between proximal sampling locations and stocked fish, a weak signal of isolation by distance, and comparatively heightened measures of effective population and genetic diversity, with substantial estimates of migration between sampling sites.

In this study, we sought to characterize the population genetic structure of Brook Trout currently found in the Upper Hudson River drainage of New York State. Specifically, we used microsatellite data from 13 previously characterized loci to address three key questions concerning Brook Trout genetic structure throughout our study region: (a) Have any of the Brook Trout in the area experienced introgression from known strains propagated by the New York State Department of Environmental Conservation (NYSDEC), and if so, is the level of introgression consistent with their proximity to stocking sites? (b) What level of population structure is present in the area, is ongoing migration occurring between tributaries, or do potential barriers to gene flow dictate population structure? (c) What do comparative levels of effective population size and genetic diversity tell us about Brook Trout genetic population structure throughout the region? To address these questions, we compared microsatellite data from our study samples to those of various stocked strains to estimate the relationship between introgression and water‐way distance from stocking sites using beta regression. We assessed population structure in the area by employing current techniques for landscape genetics, and compared measures of effective population size and genetic variation throughout the region, to elucidate site‐by‐site differences in reproductive success and genetic diversity. We then conclude this study by discussing the management implications of this work as well as how the methods used here may help to inform local conservation strategies related to species‐based conservation planning, and the identification of well‐defined genetic management units where managers and municipalities may have been historically hesitant to embrace molecular techniques.

## 2 METHODS

### 2.1 Study sites and sample collection

Upper caudal fin clippings (*n* = 337) were taken from Brook Trout in the Upper Hudson River drainage (Figure 2) in the summer of 2016 and 2017. Sample sites were chosen based on accessibility and relevance to the study goals. All fish were captured using a backpack electrofishing unit across an approximately 50–100 m stretch of stream with the exception of the fish from the Dix‐Elk watershed, which were collected in 2014 from multiple tributaries within the system and have previously been shown to be a single panmictic population (Bruce et al., 2018). Fish from all age classes were sampled across a given stretch, and genetic samples were taken from all Brook Trout sampled. Fin clippings were removed using sterile technique and stored in 95% ethanol. In addition to fin clip samples, GPS coordinates were taken for each individual sample site, as well as date and time. We also included six additional previously collected reference groups: Domestic strain Brook Trout, bred in a hatchery environment since the 1950s; Temiscamie strain Brook Trout, originally sourced from Quebec, Canada, bred in local brood ponds since the 1960s; Domestic/Temiscamie F1 hybrid strain Brook Trout, propagated yearly in a hatchery setting; and three Adirondack “heritage” strains, bred in a brood pond setting and sourced from natal waters with no stocking history, including the Little Tupper Lake strain, the Windfall Pond strain, and the Horn Lake strain. All reference groups were acquired between 2015 and 2016 from state‐administered hatcheries (*n* = 185). These populations were specifically bred for stocking purposes by the New York State Department of Environmental Conservation and are commonly used for supplementation throughout the study area, but are stocked under different circumstances. The Domestic strain and Temiscamie/Domestic F1 hybrid fish are stocked throughout the region regardless of the presence of wild reproducing Brook Trout populations, to enhance recreational angling. The three heritage strains included in the analysis (L. Tupper, Horn, and Windfall) are specifically stocked in waters that have been reclaimed (lakes and ponds where rotenone has been used to remove all fish species, prior to reintroduction), and waters where Brook Trout were previously extirpated due to acidification, but where evidence of recovery has warranted their reintroduction, while pure Temiscamie strain fish are only used to create the F1 hybrids, and do not see stocking in the study region.

### 2.2 DNA extraction and genotyping

All DNA extractions and quantifications for this study were carried out at the New York State Museum in Albany, NY. DNA for each individual fish was extracted following the tissue protocol included with the QIAGEN DNeasy tissue kit (QIAGEN, Inc., Valencia, CA). Thirteen autosomal microsatellite loci were genotyped using the same procedures for all individuals. Polymerase chain reaction (PCR) was used to amplify loci using primer pairs created specifically for Brook Trout (*SfoB52*,* SfoC24, SfoC28, SfoC38, SfoC79, SfoC86, SfoC88, SfoC113*,* SfoC115*,* SfoC129, SfoD75, SfoD91, SfoD100*; King et al., 2012). The forward primers were fluorescently labeled with HEX, FAM, or NED dye for downstream electropherogram analysis. PCR‐related methods resulted in five 20 µl multiplex PCR reactions and one single 20 µl PCR reaction for each individual fish. PCR amplification was carried out in the Research & Collections Molecular Laboratory at the New York State Museum using two Bio‐Rad T100 thermal cyclers. Fragment analysis using an internal size standard (Liz600, Applied Biosystems) was performed at the University at Albany Center for Functional Genomics using an Applied BioSystems 3730XL DNA Analyzer. The automated scoring of genotypes was carried out using the Geneious 4.0 (Biomatters Ltd.) software package. All automated genotype calls were confirmed by eye.

### 2.3 Neutrality testing and summary statistics

All 13 microsatellite loci included in this study were subjected to outlier tests of neutrality using the LOSITAN workbench (Antao, Lopes, Lopes, Beja‐Pereira, & Luikart, 2008). We ran an initial simulation to remove potentially nonneutral loci before computing the genomic mean *F*_ST_ for downstream analysis, while also running a bisection algorithm over repeated simulations to approximate a desired *F*_ST_. We treated each sampling location as a putative population employing a drift with migration model, assuming stepwise mutation across one million simulations. In addition, all samples were subjected to exact tests of Hardy–Weinberg equilibrium (Guo & Thompson, 1992) using the ARLEQUIN 3.5 software package (Excoffier & Lischer, 2010). Deviations from Hardy–Weinberg equilibrium were tested against 1,000,000 random permutations. Tests for linkage disequilibrium were also carried out between all pairs of loci, for all sample groupings, using the log‐likelihood ratio test as implemented in the program GENEPOP (Rousset, 2008).

Summary statistics, including observed (*H*_O_) and expected (*H*_E_) heterozygosity, were carried out using the ARLEQUIN 3.5 software package. In addition, the inbreeding coefficient (*F*_IS_; Weir & Cockerham, 1984) was calculated using GENEPOP and measures of allelic richness (*A*) were calculated across all sampling locations while correcting for differences in sample size using the FSTAT 2.9.3.2 software package (Goudet, 2001). Effective population sizes (*N*_e_) with 95% parametric confidence intervals were also calculated using NeEstimator v2.01 (Do et al., 2014). Finally, we performed pairwise *F*_ST_ tests (Hardy–Weinberg equilibrium not assumed), using Weir and Cockerham's unbiased estimator of *F*_ST_ (theta) using the FSTAT 2.9.3.2 software package.

### 2.4 Genetic population structure and migration

All individuals in this study were initially subjected to cluster analysis, to infer any potential introgression from hatchery fish commonly stocked in the region by the NYSDEC. Inferred ancestry for each sampling location was determined using the Bayesian clustering approach executed by the program STRUCTURE (version 2.3; Pritchard, Stephens, & Donnelly, 2000). Each sampling locality was run individually with all six strains used for supplemental stocking, assuming an admixture model with uncorrelated allele frequencies, employing an initial alpha value of 0.02 to account for differences in sample size across putative populations (Wang, 2017). We ran the analysis with a burn‐in step of 250,000 Markov Chain Monte Carlo (MCMC) iterations, followed by 500,000 MCMC iterations. Five replicates for each *K*‐value were performed assuming *K* = 4 through *K* = 8 to examine population structure across potentially different groupings.

The program CLUMPAK (Clustering Markov Packager Across K) (Kopelman, Mayzel, Jakobsson, Rosenberg, & Mayrose, 2015) was then used to permute clusters identified by STRUCTURE across independent runs for the purpose of producing bar plots for visualization as well as *Q*‐values (individual level ancestry estimates) associated with stocked strains across sampling locations. In order to assess the number of distinct groupings across all of the scenarios tested, we used the Evanno method (Evanno, Regnaut, & Goudet, 2005) of evaluating the best supported *K*‐value, as well as the value where the mean likelihood Ln(K) plateaus across increasing *K* (Earl & vonHoldt, 2012; Evanno et al., 2005) using the web program STRUCTURE HARVESTER (Earl & vonHoldt, 2012). Mean *Q*‐values associated with stocked strains from each sampling location were then plotted against their water‐way distance from the nearest stocking site to determine any correlation between introgression and proximity to State‐based stocking activities using a beta regression approach in the R package BetaReg (Ferrari & Cribari‐Neto, 2004), given that the level of hatchery ancestry for each sampling site assumes values in the standard unit interval (0, 1). Beta regression also allows for the incorporation of different link functions (log‐log link and logit link) into model selection to improve model fit in instances where extreme proportions are observed in the data (Cribari‐Neto & Zeileis, 2009). Pairwise water‐way distances between stocking sites and sampling locations were calculated using the R package RIVERDIST (Tyers, 2016). RIVERDIST is able to read river network shape files and provides tools for distance calculation along river networks. Sampling areas that appeared to be significantly influenced by stocked strains were then removed from downstream analysis to avoid potential errors in determining natural population genetic structure and migration estimates across the landscape.

Cluster analysis was then performed a second time, excluding the strains used for supplemental stocking as well as two sampling areas that exhibited comparatively heightened levels of mean hatchery ancestry (*Q*‐value ≤0.70), both exhibiting estimates associated with stocked strains at levels more than twice that of any other sampling location examined in this study. We again ran the analysis with a burn‐in step of 250,000 Markov Chain Monte Carlo (MCMC) iterations, followed by 500,000 MCMC iterations with five replicates for each *K*‐value, but this time assuming *K* = 1 through *K* = 15 to examine population structure across the wild‐collected sample set with an initial alpha value of 0.08, based on the number of putative populations in the data set. The same downstream analysis was applied to determine the number of distinct populations. In addition to cluster analysis, we examined isolation by distance (the relationship between genetic differentiation and water‐way distance) between our sampling areas. *F*_ST_ values previously produced using the program FSTAT were incorporated into Rousset's equation (Rousset, 1997) and graphed against pairwise water‐way distance, which was again calculated using the R package RIVERDIST.

Finally, recent estimates of migration between wild populations that showed minimal influence from stocked strains were estimated using the program BAYESASS (Wilson & Rannala, 2003). BAYESASS considers migrants up to two generations back and can be applied to dynamic populations that do not meet standard expectations for Hardy–Weinberg or genetic equilibrium. The Dix‐Elk population was excluded from this analysis since it was the only population not collected in the 2016–2017 timeframe. BAYESASS was run for 2,000,000 iterations with a burn‐in of 1,000,000 iterations, sampling every 200 iterations after burn‐in to estimate parameters. Ten replicate test runs using the same iterations, burn‐in, and sampling regime produced the same estimates across all comparisons, suggesting that convergence was easily achieved under these parameters.

## 3 RESULTS

Results of neutrality testing using the LOSITAN workbench suggested that all loci were selectively neutral and therefore suitable for further analysis (Supporting Information Figure S1). Tests across all sampling sites were negative for Hardy–Weinberg disequilibrium (following Bonferroni correction *α* = 0.05, initial nominal *p*‐value = 0.002). Tests for linkage disequilibrium resulted in three significant pairwise occurrences out of 1,092 pairwise tests: two between loci from Snyder Brook and one between loci from Unnamed brook 1 (following Bonferroni correction *α* = 0.05, initial nominal *p*‐value = 0.00005).

Genetic diversity indices did show variation between sampling locales, but estimates were generally in the same range across the putative populations sampled (*H_E_*: 0.51–0.69; *A*: 43.70–71.13; Table 1). The highest levels of genetic diversity were exhibited by Huntley Pond Inlet (*H*_E_ = 0.69, *A* = 71.13), whereas some of the lowest estimates of genetic diversity were attributed to both Gulf Brook (*H*_E_ = 0.56, *A* = 43.70) and Slide Brook (*H*_E_ = 0.51, *A* = 58.33). Measures of *F*_IS_ for Upper Hudson River localities all exhibited estimates slightly less than zero, whereas all other sampling areas, with the exception of Unnamed Brook 1 and Shanty Bottom Brook, exhibited *F*_IS_ measures slightly elevated from zero. Tests to determine the presence of heterozygote excesses or deficits produced no significant results. Measures of effective population size varied widely between sampling locales. Estimates of effective population size were lowest for the Blue Ledge Tributary and Vanderwhacker Brook (*N*_e_ = 14.8 and *N*_e_ = 17.3, respectively), whereas the highest measures of effective populations size were attributed to Unnamed Brook 1 and the combined Dix‐Elk Tributaries (*N*_e_ = 315.3 and *N*_e_ = 254.3, respectively).

**Table 1:** Sample sizes and summary statistics for all wild‐caught individuals. *N* = Number of specimens, *H*_E_ = Mean expected heterozygosity, *H*_O_ = Mean observed heterozygosity, *A* = Total allelic richness (based on a minimum sample size of 13 individuals), *F*_IS_ = Wright's inbreeding coefficient, *N*_e_ = Effective population size, with 95% confidence intervals

River system	Sampling location	*N*	*H*_E_	*H*_O_	*A*	*F*_IS_	*N*_e_
Hudson	Calamity Brook	29	0.63	0.64	60.39	−0.004	27.2 (19.0–42.4)
	Nate Pond Brook	26	0.68	0.70	69.67	−0.028	44.8 (29.0–85.7)
	Huntley Pond Inlet	30	0.69	0.71	71.13	−0.029	67.4 (41.8–147.1)
	Blue Ledge Tributary	16	0.63	0.64	59.88	−0.012	14.8 (9.4–26–2)
Boreas	Slide Brook	17	0.51	0.51	58.33	0.019	75.6 (27.2–∞)
	Snyder Brook	28	0.55	0.51	59.62	0.083	33.0 (21.6–58.8)
	Durgin Brook	27	0.60	0.57	67.70	0.040	114.8 (50.9–∞)
	Vanderwhacker Brook	17	0.62	0.58	60.58	0.062	17.3 (10.8–32.9)
	Unnamed Brook 1	30	0.61	0.63	64.80	−0.045	315.3 (80.3–∞)
Schroon	Dix‐Elk Tributaries	25	0.60	0.58	62.72	0.039	254.3 (67.3–∞)
	Gulf Brook	17	0.56	0.53	43.70	0.043	74.6 (26.3–∞)
	Unnamed Brook 2	13	0.61	0.57	64.00	0.065	46.4 (18.1–∞)
	Platt Creek	29	0.56	0.57	59.40	−0.012	60.7 (33.2–200.7)
	Shanty Bottom Brook	27	0.62	0.60	64.24	0.041	60.7 (33.9–185.9)

*F*_ST_ values for all sample sites are shown in Table 2. All pairwise *F*_ST_ comparisons between sampling locations were statistically significant following adjustments for multiple comparisons (following Bonferroni correction *α* = 0.05, initial nominal *p* value = 0.00055). The lowest estimated *F*_ST_ value was found between Durgin Brook and Snyder Brook (*F*_ST_ = 0.012), although this measure was followed closely by comparisons between Durgin Brook and Slide Brook (*F*_ST_ = 0.024) as well as Durgin Brook and Unnamed Brook 1 (*F*_ST_ = 0.026). Overall, sampling sites within the Boreas river system exhibited the lowest pairwise *F*_ST_ values calculated (mean *F*_ST_ =0.037). The highest *F*_ST_ values were exhibited between Huntley Pond Inlet and Slide Brook (*F*_ST_ = 0.184) followed by Huntley Pond Inlet and Gulf Brook (*F*_ST_ = 0.179).

**Table 2:** Pairwise *F*_ST_ values between all sample sites, calculated using Weir and Cockerham's unbiased estimator of *F*_ST_ (theta)

	Calamity	Nate	Huntley	Blue Ledge	Slide	Snyder	Durgin	Vanderwhacker	Unnamed 1	Dix‐Elk	Gulf	Unnamed 2	Platt	Shanty Bottom
Calamity	0	0.078	0.105	0.098	0.104	0.108	0.054	0.088	0.082	0.079	0.134	0.032	0.047	0.111
Nate		0	0.035	0.051	0.141	0.118	0.083	0.112	0.100	0.111	0.125	0.062	0.081	0.072
Huntley			0	0.050	0.184	0.159	0.11	0.146	0.135	0.138	0.179	0.109	0.124	0.086
Blue Ledge				0	0.169	0.155	0.108	0.151	0.119	0.116	0.150	0.094	0.109	0.069
Slide					0	0.052	0.024	0.068	0.041	0.079	0.105	0.074	0.087	0.147
Snyder						0	0.012	0.030	0.049	0.072	0.103	0.061	0.086	0.121
Durgin							0	0.021	0.026	0.036	0.082	0.031	0.051	0.090
Vanderwhacker								0	0.047	0.064	0.095	0.079	0.079	0.116
Unnamed 1									0	0.059	0.094	0.048	0.056	0.105
Dix‐Elk										0	0.084	0.045	0.043	0.102
Gulf											0	0.083	0.054	0.145
Unnamed 2												0	0.016	0.090
Platt													0	0.116
Shanty Bottom														0

**Figure 3:**
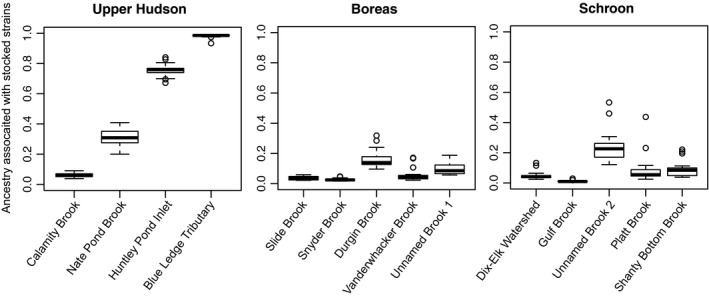
Box‐and‐whisker plots showing ancestry estimates for each sampling location associated with stocked fish across three river systems, calculated using *Q*‐values produced by the program STRUCTURE and permuted using CLUMPAK. The horizontal line in the box marks the median, the box edges (hinges) the first and third quartile. The interquartile range within the box includes the central 50% of the values. The whiskers show the range of observed values that are not within the first and third quartile but not further away than 1.5 times the interquartile range from the hinges. Values farther than three times the interquartile range from the next hinge are marked by open circles

**Figure 4:**
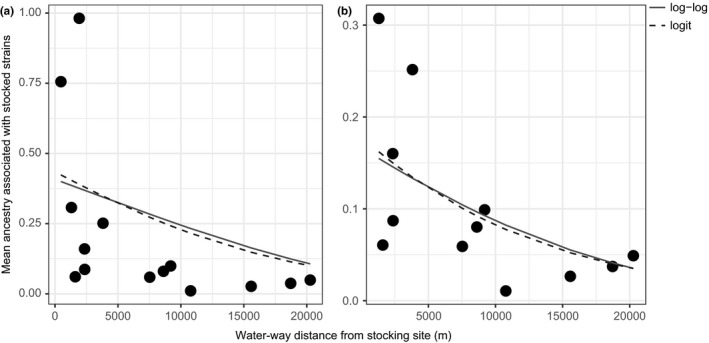
Mean level of ancestry associated with stocked strains (see Figure 2) plotted against distance to the nearest proximal stocking site (see Figure 1) for (a) all sampling locations examined in this study, and (b) excluding outliers Huntley Pond Inlet and Blue Ledge Tributary. Points on the chart represent sampling locations, and the fitted curves correspond to beta regressions gy_loglog with log‐log link (solid) and gy_logit with logit link (dashed). Both treatments are statistically significant (a: *p* = 0.0047, pseudo *R* ^2^ = 0.34; b: *p* = 0.0137, pseudo *R* ^2^ = 0.39)

For both the Δ*K* and Ln(*K*) measures produced by STRUCTURE HARVESTER for the runs that included the stocked strains, all sampling locations were identified as genetically distinct from the supplementation strains (*K* = 6). Bar plots produced by STRUCTURE and processed with CLUMPAK, which examined each sampling site individually with these strains, suggested that the many of the sampled individuals show comparatively low estimates of introgression from long‐term stocking in the area, exhibiting minimal signs of admixture associated with the New York State stocked strains compared to fish sampled from Huntley Pond Inlet and the Blue Ledge Tributary (Figure 3, Supporting Information Figure S2). Individuals from Huntley Pond Inlet and the Blue Ledge Tributary were the exceptions, exhibiting mean ancestry estimates associated with stocked strains at levels greater than 70% (*Q*‐value ≥0.70), greater than twice that of any other sample site examined; these individuals were therefore removed from downstream analyses that examined genetic structure between wild fish, to exclude possible bias associated with human‐mediated translocations. Gulf Brook and Snyder Brook exhibited the lowest level of ancestry associated with the New York stocked strains (*Q*‐value ≤0.03). All other populations exhibited mean hatchery ancestry at levels that ranged anywhere between 3% and 35% (*Q*‐value; 0.03–0.35), with the majority of individuals falling in the 3%–10% range (*Q*‐value; 0.03–0.10). Beta regression analysis suggested a significant relationship between hatchery ancestry and water‐way distance to the nearest stocking site (Figure 4; Supporting Information Table S4), regardless of the inclusion of the two outlier sites which exhibited heightened levels of hatchery ancestry (Huntley Pond Inlet and the Blue Ledge Tributary).

**Figure 5:**
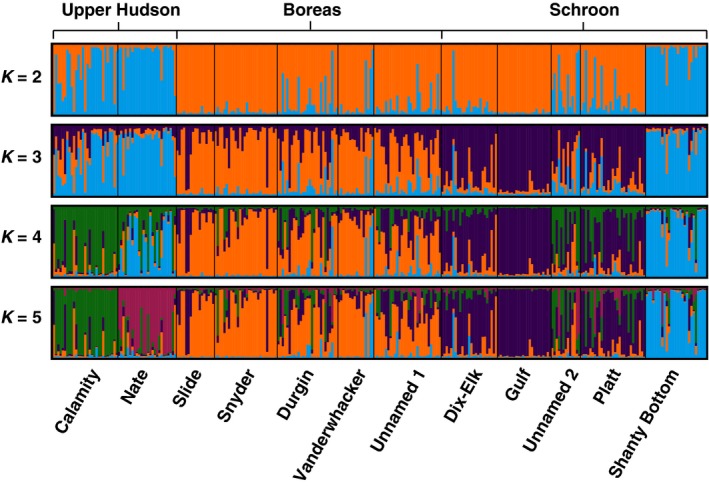
STRUCTURE bar plots across all sampling locations, excluding Huntley Pond Inlet and the Blue Ledge Tributary for *K* values 2 through 5. Each vertical line represents an individual, and colors represent their inferred ancestry from *K* ancestral populations. Labels at the top correspond to the major river systems into which the respective tributaries drain

**Figure 6:**
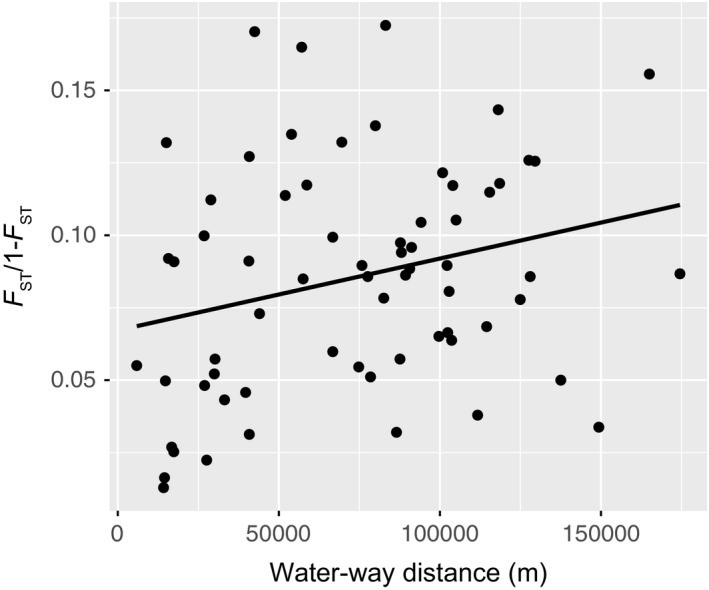
Isolation‐by‐distance plot showing results of a Mantel test performed to identify correlation between natural genetic differentiation and the water‐way distance between sampling locations (Huntley Pond Inlet and Blue Ledge Tributary excluded). Though weak, a positive correlation was found to be statistically significant (*p* = 0.034, *R* ^2^ = 0.0687)

When comparing the remaining sampling sites (after removal of Huntley Pond Inlet and the Blue Ledge Tributary populations) to each other, the Δ*K* measure and the Ln(*K*) measure produced conflicting results (Supporting Information Figure S3); therefore, the succession of bar plots in this range was examined simultaneously (Figure 5). High levels of admixture were exhibited across all sampling sites at all *K*‐values, with the exception of Gulf Brook. When examining the *K* = 2 bar plot, we found that the Upper Hudson River samples group with the Shanty Bottom Brook population in the Schroon system, while all other populations group together with some level of reciprocal admixture between groupings. With increasing *K*‐values, the other Schroon sampling sites break out together, followed by the individual Hudson sampling locales breaking out on their own. The Boreas River system remains highly admixed throughout, as do the majority of the Schroon samples (with the exception of Gulf Brook). These results indicate weak population structure at the level of the individual river system and sampling sites, with substantial mixing between individuals at sampling sites both within and between river systems. The isolation‐by‐distance plot suggests a statistically significant linear correlation between genetic differentiation and water‐way distance, although this correlation is somewhat weak, with water‐way distance explaining approximately seven percent of the variation in genetic differentiation between sample sites (Figure 6).

**Figure 7:**
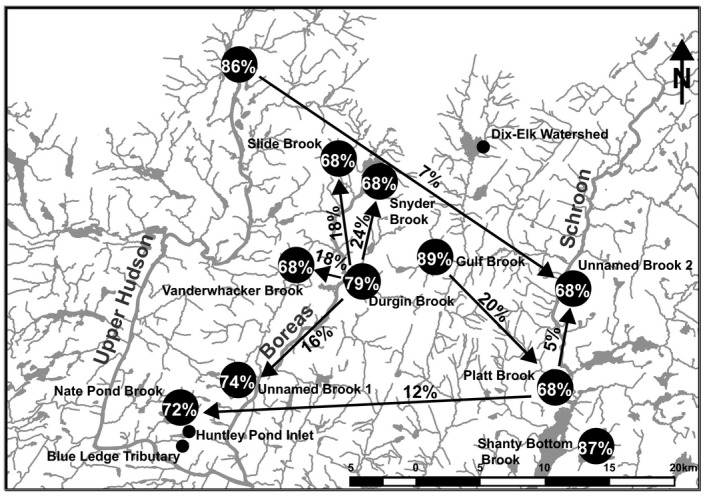
Estimated migration between sampling sites. Numbers above arrows indicate the fraction of individuals from population (a) that are derived from population (b) (m), while numbers within the circles denote the proportion of nonimmigrants (*NM*) within populations. For clarity of presentation, *M*‐values <5% are not shown

Migration estimates produced by BAYESASS mirror the results of the STRUCTURE analysis (Figure 7, Supporting Information Table S5). While ongoing migration (*M*) was detected to some extent across all sampling sites, it was estimated to be the highest within the Boreas River system (*M* = 16%–24%). Estimates of the number of nonmigrants (*NM*) for each sampling site within this region were also comparatively low (*NM* = 68%–74%). The number of nonmigrants exhibited by the Gulf Brook populations was the highest (*NM* = 89%), while both Shanty Bottom Brook and Calamity Brook produced similarly high measures (*NM* = 87% and 86%, respectively). Migration estimates were generally much lower between river systems than within, consistent with the admixture patterns produced by STRUCTURE.

## 4 DISCUSSION

Understanding how anthropogenic influences have affected the natural genetic structure of communities across the landscape is key to making responsible decisions about the effective management of those species, especially if they are in a state of decline (Laikre, Palm, & Ryman, 2005; Schwartz, Luikart, & Waples, 2007). As human‐induced habitat alterations continue to alter the genetic structure of wild populations, informed consideration should be given to how these challenges may be influencing the livelihood of wild‐reproducing fish species. Thus, it has become critical to gain a clear understanding of how fish that live in interconnected river systems breed and interact.

In this study, we have demonstrated that the Brook Trout currently inhabiting the Upper Hudson drainage in New York State (a) exhibit varying degrees of introgression from state‐based stocking activities, which shows a significant positive relationship with proximity to stocking sites; (b) exhibit weak genetic population structure at the level of individual tributaries as well as the larger river systems where they are found; (c) are experiencing ongoing migration at least partially influenced by geographic distance; and (d) demonstrate comparatively similar measures of genetic diversity but varied measures of effective population size, based on sampling location.

When comparing the genetic structure of fish collected on a site‐by‐site basis with the strains currently stocked by the NYSDEC, it is apparent that although introgression from stocked fish is minimal in most cases, it is present and is in discernable amounts throughout the study area. In addition, admixture estimates produced using the program STRUCTURE attributed this introgression almost entirely to Domestic strain fish, Temiscamie strain fish, or in most cases some combination of the two. This is an important finding because it suggests that the Adirondack “heritage” strains used for stocking in the region are not readily interbreeding with wild fish stocks. Nevertheless, the Temiscamie/Domestic F1 hybrid fish is the primary fish used for supplementation on top of wild reproducing fish in this region, so whether this introgression is a product of a selective advantage over the other stocked strains, or based on circumstantial stocking practices is ultimately unclear. Unfortunately, comprehensive, historical stocking data for this region were not readily available, and supplemental stocking has been occurring in this watershed for decades, so any link between stocking intensity and introgression would be speculative. Nevertheless, we were able to detect a significant relationship between the level of introgression from stocked fish and water‐way distance to known stocking sites; although this model did not explicitly explain the level of introgression for all individuals sampled, these results do suggest distance from stocking sites may be one of many factors contributing to the level of hybridization observed. When taking all of these findings into context, the introgressive hybridization elucidated here has substantial implications from a management standpoint, especially if the goal of stocking is to provide opportunities for anglers while limiting genetic impacts on native, naturally reproducing populations. The two populations that showed the highest levels of putative hatchery ancestry (Huntley Pond Inlet and the Blue Ledge Tributary) are also in very close proximity to each other, as well as Huntley Pond, a reclaimed trout pond, which is supplemented with Temiscamie/Domestic hybrids on an annual basis (NYSDEC, 2010). These results suggest that the fish currently found in that area retain a discernable amount of ancestry linked to supplementation events.

Our findings also suggest that when an area is stocked, the stocked fish have the potential to move into, and mix with, fish in neighboring tributaries. None of the fish that exhibited hatchery ancestry in this study were determined to be purebred stocked fish; all stocked ancestry was attributed to admixed individuals. This was a surprising finding, for which the reasons are unclear. The main stems of the Boreas, Schroon, and Upper Hudson rivers are all regularly stocked with hatchery‐bred fish from both State and local municipalities (NYSDEC, 2017). Despite the widespread stocking of these rivers, the majority of Brook Trout populations sampled from directly connected tributaries to these systems showed surprisingly low amounts of ancestry related to stocked Brook Trout strains, and no evidence of recently stocked fish. This would suggest that stocked fish in these areas are currently making, at most, a limited contribution to wild reproduction in the region.

Admixture estimates and estimates of migration for the relatively nonintrogressed wild‐caught samples are analogous and suggest ongoing migration both within and to a lesser extent between river systems. This is an important finding, as relatively little work has been done with this species to determine levels of migration in such a network (but see Curry, Sparks, & Sande, 2002). Given the heightened levels of movement between nearby sampling sites, especially in the Boreas system, considerations related to maintaining connectivity and, by extension, genetic integrity are warranted. Estimates of migration at levels greater than 5% are all unidirectional. This may suggest that most if not all areas exhibit landscape features potentially influencing gene flow in one direction or another. Nevertheless, the overall remoteness and inaccessibility of the area makes obtaining landscape level habitat information between most sampling sites unrealistic. More recent studies that have looked at genetic population structure in dendritic stream networks have suggested that Brook Trout in these systems exhibit relatively little movement (Hudy et al., 2010; Kanno et al., 2011). The findings of this study suggest that Brook Trout population dynamics in larger interconnected systems may be far more complex. The results of the isolation by distance analysis also found a significant relationship between physical distance and genetic differentiation, although this trend is weak. Given that there are myriad factors including (but not limited to) waterfalls, elevation gradients, pitched culverts, and the presence of nonnative competitors potentially influencing connectivity and gene flow in these systems, these results demonstrate that water‐way distance remains a significant contributor to population structure in the wild‐collected fish of this area.

When our findings regarding ongoing migration are viewed in conjunction with the results of pairwise *F*_ST_ analysis and genetic diversity indices, we see that several populations, such as Gulf Brook and Shanty Bottom Brook, seem to be somewhat cut off from neighboring sites. Gulf Brook in particular showed some of the lowest diversity, admixture, and migration estimates in the study. These are indications that there may be barriers to two‐way gene flow, allowing limited movement into these tributaries. In the case of Gulf Brook, this may be a steep elevation gradient in the form of a waterfall or a pitched culvert, whereas in the case of Shanty Bottom Brook, Schroon Lake, into which this brook drains, may be difficult to traverse given its large size and occupancy by nonnative competitors such as bass and perch (Odell, 1932). Shanty Bottom Brook also retains genetic structure more similar to the Upper Hudson River sites then to the other Schroon River locations, but whether the reasons for this are based on putative barriers to gene flow, long held demographic differences based on postglacial recolonization, historic undocumented translocations, or some combination of the above is unclear. Regardless of the reasons why, these populations may act as sinks for unique genetic variation, given that they have likely seen relatively little outside influence from either contemporary or historically stocked fish, or the larger Brook Trout assemblage inhabiting the area. Given that detailed habitat data for this area are widely unavailable, employing genetic techniques to identify regions where barriers to gene flow may be present is a logical starting point to mapping genetic diversity across the landscape and identifying areas where dispersal may be limited.

Genetic diversity indices (*H*_E_, *A*, and *F*_IS_) were relatively similar across all sampling sites, while estimates of effective population size (*N*_e_) varied widely. The comparatively large effective population size of the combined Dix‐Elk tributaries (*N*_e_ = 254.3) may be a result of sampling across a comparatively wider range (i.e., sampling inconsistencies), but the comparatively large effective population size for Unnamed Brook 1 and Durgin Brook (*N*_e_ = 315.3 and 114.8 respectively), taken into consideration with the migration estimates for these areas, suggests that these tributaries (especially Durgin Brook) may play a central role in reproduction and dispersal throughout the system. In addition, confidence intervals for effective population size estimates across all sample sites were large in most cases, with upper limits in the infinity range for many areas, suggesting open populations not restricted to the sampled stream stretch.

As human activities continue to transform habitats across the Northeastern United States, identifying and understanding how hydrology affects dispersal and gene flow are critical to preserving an aquatic species’ ability to adapt. In addition, understanding how human‐mediated transference of species affects native assemblages is paramount to preserving natural genetic population structure, especially that of indigenous populations that possess local adaptations. In this study, we utilized genetic markers to assess intraspecific interactions and migration in a complex, wide‐ranging system, where a traditional radio‐tag or mark and recapture study would have been logistically challenging, potentially unable to answer all of the study questions, and likely more costly. There is currently a general consensus among researchers and fisheries managers regarding the need for species‐focused conservation plans, although the role of molecular applications in such plans continues to be disputed (Allendorf, Hohenlohe, & Luikart, 2010; Garcia de Leaniz et al., 2007). Nevertheless, we hope that the methods presented here may offer insight into how genetic analyses can answer complex questions related to species conservation not only in New York, but in any location where aquatic species of local conservation concern face similar circumstances and challenges.

## **AUTHOR CONTRIBUTIONS**

The authors made the following contributions to the work presented here. S.B. conceived the project; S.B., J.W. developed the project; S.B., J.W. participated in fieldwork; S.B. performed all laboratory work from DNA extraction to genotyping; S.B. performed the statistical analysis with input from J.W.; S.B. wrote the paper with input from J.W.

## DATA ACCESSIBILITY

GPS coordinates for all sampling locations and microsatellite genotype data from this study are freely available to all interested parties through the Dryad database. https://doi.org/10.5061/dryad.c68b045.
