# Supplementary material for: Estimates of gene flow and dispersal in wild riverine Brook Trout (Salvelinus fontinalis) populations reveal ongoing migration and introgression from stocked fish
Source: Ecol Evol. 2018 Nov 14;8(23):11410–22. doi: 10.1002/ece3.4556 (PMC6303771; doi:10.1002/ece3.4556)
Supplement: Supplementary file 1 [file ECE3-8-11410-s001.docx]

**Supplemental Information for:**

**Estimates of gene flow and dispersal in wild riverine Brook Trout (*Salvelinus fontinalis*) populations reveal ongoing migration and introgression from stocked fish**

Spencer A. Bruce, Jeremy J. Wright

**Table of Contents:**

| **Supplementary 1** | Page 1 |
| --- | --- |
| **Supplementary 2** | Page 2 |
| **Supplementary 3** | Page 3 |
| **Supplementary 4** | Page 4 |
| **Supplementary 5** | Page 5 |


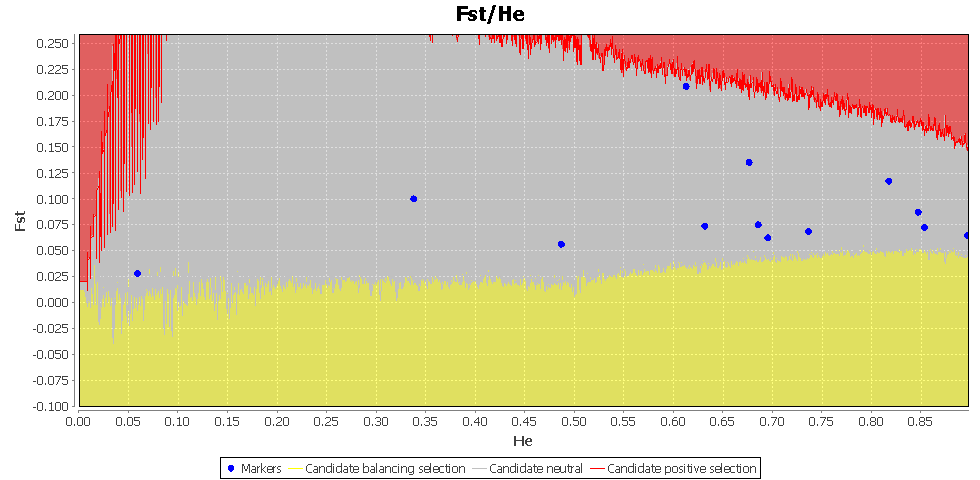


Supplemental 1: Neutrality testing output from LOSITAN using the *F*_ST_ outlier method to detect selection across all sample sites. Heterozygosity was plotted against *F_ST_* for all 13 microsatellite loci. Ranges of values for balancing selection (colored in yellow), positive selection (colored in red) and neutrality (colored in grey) were identified. All 13 microsatellite loci fell into the acceptable range of neutrality

Supplemental 2: STRUCTURE bar plots for stocked strains and individual sampling locations (K = 6). Each vertical line represents an individual, and colors represent their inferred ancestry from K ancestral populations.

Supplemental 3: ΔK and average mean likelihood Ln(K) values for STRUCTURE plots (Figure 4), ΔK suggesting two populations, and Ln(K) suggesting six.

|  | Cala-mity | Nate | Huntley | Blue Ledge | Slide | Snyder | Durgin | Vander-whacker | Un-named 1 | Dix-Elk | Gulf | Un-named 2 | Platt | Shanty Bottom |
| --- | --- | --- | --- | --- | --- | --- | --- | --- | --- | --- | --- | --- | --- | --- |
| Calamity | 0 | 57.52 | 55.86 | 55.46 | 103.97 | 100.85 | 87.62 | 87.76 | 75.79 | 174.52 | 165.02 | 149.33 | 137.59 | 129.54 |
| Nate |  | 0 | 8.94 | 8.54 | 57.07 | 53.91 | 40.69 | 40.83 | 28.87 | 127.60 | 118.10 | 102.41 | 90.67 | 82.63 |
| Huntley |  |  | 0 | 2.39 | 54.20 | 51.06 | 37.82 | 37.96 | 26.00 | 124.73 | 115.23 | 99.54 | 87.80 | 79.75 |
| Blue Ledge |  |  |  | 0 | 53.80 | 50.67 | 37.42 | 37.56 | 25.59 | 124.33 | 114.83 | 99.13 | 87.40 | 79.34 |
| Slide |  |  |  |  | 0 | 5.90 | 17.37 | 43.99 | 33.12 | 128.09 | 118.59 | 102.89 | 91.157 | 83.11 |
| Snyder |  |  |  |  |  | 0 | 14.23 | 40.84 | 29.98 | 124.95 | 115.44 | 99.75 | 88.01 | 79.97 |
| Durgin |  |  |  |  |  |  | 0 | 27.60 | 16.74 | 111.71 | 102.21 | 86.51 | 74.78 | 66.73 |
| Vanderwhacker |  |  |  |  |  |  |  | 0 | 14.83 | 114.51 | 105.01 | 89.32 | 77.58 | 69.53 |
| Unnamed 1 |  |  |  |  |  |  |  |  | 0 | 103.65 | 94.15 | 78.45 | 66.72 | 58.67 |
| Dix-Elk |  |  |  |  |  |  |  |  |  | 0 | 15.70 | 26.93 | 39.72 | 51.98 |
| Gulf |  |  |  |  |  |  |  |  |  |  | 0 | 17.43 | 30.22 | 42.48 |
| Unnamed 2 |  |  |  |  |  |  |  |  |  |  |  | 0 | 14.53 | 26.79 |
| Platt |  |  |  |  |  |  |  |  |  |  |  |  | 0 | 15.05 |
| Shanty Bottom |  |  |  |  |  |  |  |  |  |  |  |  |  | 0 |

Supplemental 4: Pairwise water-way distance (km) between all sample sites, calculated using the R package riverdist.

| m[0][0]: | 0.8627(0.0326) | m[0][1]: | 0.0094(0.0093) | m[0][2]: | 0.0083(0.0081) | m[0][3]: | 0.0084(0.0083) |
| --- | --- | --- | --- | --- | --- | --- | --- |
| m[1][0]: | 0.0290(0.0292) | m[1][1]: | 0.7193(0.0504) | m[1][2]: | 0.0090(0.0089) | m[1][3]: | 0.0092(0.0089) |
| m[2][0]: | 0.0120(0.0117) | m[2][1]: | 0.0120(0.0116) | m[2][2]: | 0.6786(0.0116) | m[2][3]: | 0.0118(0.0113) |
| m[3][0]: | 0.0100(0.0095) | m[3][1]: | 0.0086(0.0084) | m[3][2]: | 0.0085(0.0083) | m[3][3]: | 0.6755(0.0085) |
| m[4][0]: | 0.0407(0.0285) | m[4][1]: | 0.0124(0.0120) | m[4][2]: | 0.0088(0.0087) | m[4][3]: | 0.0089(0.0085) |
| m[5][0]: | 0.0126(0.0123) | m[5][1]: | 0.0125(0.0120) | m[5][2]: | 0.0124(0.0119) | m[5][3]: | 0.0124(0.0120) |
| m[6][0]: | 0.0138(0.0163) | m[6][1]: | 0.0107(0.0107) | m[6][2]: | 0.0082(0.0078) | m[6][3]: | 0.0081(0.0079) |
| m[7][0]: | 0.0100(0.0095) | m[7][1]: | 0.0097(0.0092) | m[7][2]: | 0.0095(0.0091) | m[7][3]: | 0.0092(0.0091) |
| m[8][0]: | 0.0679(0.0429) | m[8][1]: | 0.0162(0.0163) | m[8][2]: | 0.0138(0.0132) | m[8][3]: | 0.0141(0.0135) |
| m[9][0]: | 0.0188(0.0153) | m[9][1]: | 0.0090(0.0088) | m[9][2]: | 0.0089(0.0087) | m[9][3]: | 0.0087(0.0085) |
| m[10][0]: | 0.0205(0.0163) | m[10][1]: | 0.0113(0.0105) | m[10][2]: | 0.0091(0.0092) | m[10][3]: | 0.0087(0.0082) |
| m[0][4]: | 0.0352(0.0267) | m[0][5]: | 0.0083(0.0083) | m[0][6]: | 0.0244(0.0243) | m[0][7]: | 0.0138(0.0125) |
| m[1][4]: | 0.0214(0.0204) | m[1][5]: | 0.0093(0.0091) | m[1][6]: | 0.0484(0.0533) | m[1][7]: | 0.0155(0.0152) |
| m[2][4]: | 0.1773(0.0463) | m[2][5]: | 0.0118(0.0114) | m[2][6]: | 0.0243(0.0373) | m[2][7]: | 0.0363(0.0201) |
| m[3][4]: | 0.2369(0.0233) | m[3][5]: | 0.0085(0.0083) | m[3][6]: | 0.0099(0.0097) | m[3][7]: | 0.0161(0.0122) |
| m[4][4]: | 0.7853(0.0588) | m[4][5]: | 0.0088(0.0088) | m[4][6]: | 0.0492(0.0566) | m[4][7]: | 0.0351(0.0250) |
| m[5][4]: | 0.1797(0.0363) | m[5][5]: | 0.6793(0.0125) | m[5][6]: | 0.0165(0.0173) | m[5][7]: | 0.0140(0.0135) |
| m[6][4]: | 0.1586(0.0900) | m[6][5]: | 0.0084(0.0081) | m[6][6]: | 0.7417(0.0843) | m[6][7]: | 0.0187(0.0199) |
| m[7][4]: | 0.0255(0.0187) | m[7][5]: | 0.0094(0.0090) | m[7][6]: | 0.0125(0.0120) | m[7][7]: | 0.8854(0.0296) |
| m[8][4]: | 0.0360(0.0269) | m[8][5]: | 0.0136(0.0130) | m[8][6]: | 0.0453(0.0487) | m[8][7]: | 0.0464(0.0290) |
| m[9][4]: | 0.0162(0.0151) | m[9][5]: | 0.0088(0.0084) | m[9][6]: | 0.0282(0.0252) | m[9][7]: | 0.2009(0.0353) |
| m[10][4]: | 0.0251(0.0190) | m[10][5]: | 0.0086(0.0085) | m[10][6]: | 0.0143(0.0141) | m[10][7]: | 0.0149(0.0124) |
| m[0][8]: | 0.0086(0.0085) | m[0][9]: | 0.0119(0.0113) | m[0][10]: | 0.0091(0.0087) |  |  |
| m[1][8]: | 0.0093(0.0091) | m[1][9]: | 0.1184(0.0912) | m[1][10]: | 0.0112(0.0109) |  |  |
| m[2][8]: | 0.0119(0.0116) | m[2][9]: | 0.0120(0.0116) | m[2][10]: | 0.0119(0.0114) |  |  |
| m[3][8]: | 0.0087(0.0084) | m[3][9]: | 0.0087(0.0083) | m[3][10]: | 0.0086(0.0083) |  |  |
| m[4][8]: | 0.0092(0.0087) | m[4][9]: | 0.0280(0.0204) | m[4][10]: | 0.0136(0.0127) |  |  |
| m[5][8]: | 0.0125(0.0120) | m[5][9]: | 0.0132(0.0127) | m[5][10]: | 0.0349(0.0228) |  |  |
| m[6][8]: | 0.0083(0.0082) | m[6][9]: | 0.0110(0.0108) | m[6][10]: | 0.0125(0.0119) |  |  |
| m[7][8]: | 0.0093(0.0089) | m[7][9]: | 0.0101(0.0094) | m[7][10]: | 0.0093(0.0095) |  |  |
| m[8][8]: | 0.6806(0.0132) | m[8][9]: | 0.0522(0.0273) | m[8][10]: | 0.0139(0.0134) |  |  |
| m[9][8]: | 0.0090(0.0088) | m[9][9]: | 0.6830(0.0145) | m[9][10]: | 0.0086(0.0085) |  |  |
| m[10][8]: | 0.0089(0.0090) | m[10][9]: | 0.0125(0.0118) | m[10][10]: | 0.8662(0.0287) |  |  |

Supplemental 5: Pairwise migration rates for all sampling sites produced using the program BAYESASS. Note that m[i][j] is the fraction of individuals in population i that are migrants derived from population j (per generation). Next are the mean posterior estimates (and standard errors) for each sample site. Sample sites are coded as follows; 0: Calamity Brook, 1: Nate Brook, 2: Slide Brook, 3: Snyder Brook, 4: Durgin Brook, 5: Vanderwhacker Brook, 6: Unnamed Brook 1, 7: Gulf Brook, 8: Unnamed Brook 2, 9: Platt Brook, 10: Shanty Bottom Brook.
