# Supplementary material for: Estimates of gene flow and dispersal in wild riverine Brook Trout (Salvelinus fontinalis) populations reveal ongoing migration and introgression from stocked fish
Source: Ecol Evol. 2018 Nov 14;8(23):11410–22. doi: 10.1002/ece3.4556 (PMC6303771; doi:10.1002/ece3.4556)
Supplement: Supplementary file 5 [file ECE3-8-11410-s005.docx]

|  | Cala-mity | Nate | Huntley | Blue Ledge | Slide | Snyder | Durgin | Vander-whacker | Un-named 1 | Dix-Elk | Gulf | Un-named 2 | Platt | Shanty Bottom |
| --- | --- | --- | --- | --- | --- | --- | --- | --- | --- | --- | --- | --- | --- | --- |
| Calamity | 0 | 57.52 | 55.86 | 55.46 | 103.97 | 100.85 | 87.62 | 87.76 | 75.79 | 174.52 | 165.02 | 149.33 | 137.59 | 129.54 |
| Nate |  | 0 | 8.94 | 8.54 | 57.07 | 53.91 | 40.69 | 40.83 | 28.87 | 127.60 | 118.10 | 102.41 | 90.67 | 82.63 |
| Huntley |  |  | 0 | 2.39 | 54.20 | 51.06 | 37.82 | 37.96 | 26.00 | 124.73 | 115.23 | 99.54 | 87.80 | 79.75 |
| Blue Ledge |  |  |  | 0 | 53.80 | 50.67 | 37.42 | 37.56 | 25.59 | 124.33 | 114.83 | 99.13 | 87.40 | 79.34 |
| Slide |  |  |  |  | 0 | 5.90 | 17.37 | 43.99 | 33.12 | 128.09 | 118.59 | 102.89 | 91.157 | 83.11 |
| Snyder |  |  |  |  |  | 0 | 14.23 | 40.84 | 29.98 | 124.95 | 115.44 | 99.75 | 88.01 | 79.97 |
| Durgin |  |  |  |  |  |  | 0 | 27.60 | 16.74 | 111.71 | 102.21 | 86.51 | 74.78 | 66.73 |
| Vanderwhacker |  |  |  |  |  |  |  | 0 | 14.83 | 114.51 | 105.01 | 89.32 | 77.58 | 69.53 |
| Unnamed 1 |  |  |  |  |  |  |  |  | 0 | 103.65 | 94.15 | 78.45 | 66.72 | 58.67 |
| Dix-Elk |  |  |  |  |  |  |  |  |  | 0 | 15.70 | 26.93 | 39.72 | 51.98 |
| Gulf |  |  |  |  |  |  |  |  |  |  | 0 | 17.43 | 30.22 | 42.48 |
| Unnamed 2 |  |  |  |  |  |  |  |  |  |  |  | 0 | 14.53 | 26.79 |
| Platt |  |  |  |  |  |  |  |  |  |  |  |  | 0 | 15.05 |
| Shanty Bottom |  |  |  |  |  |  |  |  |  |  |  |  |  | 0 |

Supplemental 4: Pairwise water-way distance (km) between all sample sites, calculated using the R package riverdist.
