# Supplementary material for: Estimates of gene flow and dispersal in wild riverine Brook Trout (Salvelinus fontinalis) populations reveal ongoing migration and introgression from stocked fish
Source: Ecol Evol. 2018 Nov 14;8(23):11410–22. doi: 10.1002/ece3.4556 (PMC6303771; doi:10.1002/ece3.4556)
Supplement: Supplementary file 6 [file ECE3-8-11410-s006.docx]

| m[0][0]: | 0.8627(0.0326) | m[0][1]: | 0.0094(0.0093) | m[0][2]: | 0.0083(0.0081) | m[0][3]: | 0.0084(0.0083) |
| --- | --- | --- | --- | --- | --- | --- | --- |
| m[1][0]: | 0.0290(0.0292) | m[1][1]: | 0.7193(0.0504) | m[1][2]: | 0.0090(0.0089) | m[1][3]: | 0.0092(0.0089) |
| m[2][0]: | 0.0120(0.0117) | m[2][1]: | 0.0120(0.0116) | m[2][2]: | 0.6786(0.0116) | m[2][3]: | 0.0118(0.0113) |
| m[3][0]: | 0.0100(0.0095) | m[3][1]: | 0.0086(0.0084) | m[3][2]: | 0.0085(0.0083) | m[3][3]: | 0.6755(0.0085) |
| m[4][0]: | 0.0407(0.0285) | m[4][1]: | 0.0124(0.0120) | m[4][2]: | 0.0088(0.0087) | m[4][3]: | 0.0089(0.0085) |
| m[5][0]: | 0.0126(0.0123) | m[5][1]: | 0.0125(0.0120) | m[5][2]: | 0.0124(0.0119) | m[5][3]: | 0.0124(0.0120) |
| m[6][0]: | 0.0138(0.0163) | m[6][1]: | 0.0107(0.0107) | m[6][2]: | 0.0082(0.0078) | m[6][3]: | 0.0081(0.0079) |
| m[7][0]: | 0.0100(0.0095) | m[7][1]: | 0.0097(0.0092) | m[7][2]: | 0.0095(0.0091) | m[7][3]: | 0.0092(0.0091) |
| m[8][0]: | 0.0679(0.0429) | m[8][1]: | 0.0162(0.0163) | m[8][2]: | 0.0138(0.0132) | m[8][3]: | 0.0141(0.0135) |
| m[9][0]: | 0.0188(0.0153) | m[9][1]: | 0.0090(0.0088) | m[9][2]: | 0.0089(0.0087) | m[9][3]: | 0.0087(0.0085) |
| m[10][0]: | 0.0205(0.0163) | m[10][1]: | 0.0113(0.0105) | m[10][2]: | 0.0091(0.0092) | m[10][3]: | 0.0087(0.0082) |
| m[0][4]: | 0.0352(0.0267) | m[0][5]: | 0.0083(0.0083) | m[0][6]: | 0.0244(0.0243) | m[0][7]: | 0.0138(0.0125) |
| m[1][4]: | 0.0214(0.0204) | m[1][5]: | 0.0093(0.0091) | m[1][6]: | 0.0484(0.0533) | m[1][7]: | 0.0155(0.0152) |
| m[2][4]: | 0.1773(0.0463) | m[2][5]: | 0.0118(0.0114) | m[2][6]: | 0.0243(0.0373) | m[2][7]: | 0.0363(0.0201) |
| m[3][4]: | 0.2369(0.0233) | m[3][5]: | 0.0085(0.0083) | m[3][6]: | 0.0099(0.0097) | m[3][7]: | 0.0161(0.0122) |
| m[4][4]: | 0.7853(0.0588) | m[4][5]: | 0.0088(0.0088) | m[4][6]: | 0.0492(0.0566) | m[4][7]: | 0.0351(0.0250) |
| m[5][4]: | 0.1797(0.0363) | m[5][5]: | 0.6793(0.0125) | m[5][6]: | 0.0165(0.0173) | m[5][7]: | 0.0140(0.0135) |
| m[6][4]: | 0.1586(0.0900) | m[6][5]: | 0.0084(0.0081) | m[6][6]: | 0.7417(0.0843) | m[6][7]: | 0.0187(0.0199) |
| m[7][4]: | 0.0255(0.0187) | m[7][5]: | 0.0094(0.0090) | m[7][6]: | 0.0125(0.0120) | m[7][7]: | 0.8854(0.0296) |
| m[8][4]: | 0.0360(0.0269) | m[8][5]: | 0.0136(0.0130) | m[8][6]: | 0.0453(0.0487) | m[8][7]: | 0.0464(0.0290) |
| m[9][4]: | 0.0162(0.0151) | m[9][5]: | 0.0088(0.0084) | m[9][6]: | 0.0282(0.0252) | m[9][7]: | 0.2009(0.0353) |
| m[10][4]: | 0.0251(0.0190) | m[10][5]: | 0.0086(0.0085) | m[10][6]: | 0.0143(0.0141) | m[10][7]: | 0.0149(0.0124) |
| m[0][8]: | 0.0086(0.0085) | m[0][9]: | 0.0119(0.0113) | m[0][10]: | 0.0091(0.0087) |  |  |
| m[1][8]: | 0.0093(0.0091) | m[1][9]: | 0.1184(0.0912) | m[1][10]: | 0.0112(0.0109) |  |  |
| m[2][8]: | 0.0119(0.0116) | m[2][9]: | 0.0120(0.0116) | m[2][10]: | 0.0119(0.0114) |  |  |
| m[3][8]: | 0.0087(0.0084) | m[3][9]: | 0.0087(0.0083) | m[3][10]: | 0.0086(0.0083) |  |  |
| m[4][8]: | 0.0092(0.0087) | m[4][9]: | 0.0280(0.0204) | m[4][10]: | 0.0136(0.0127) |  |  |
| m[5][8]: | 0.0125(0.0120) | m[5][9]: | 0.0132(0.0127) | m[5][10]: | 0.0349(0.0228) |  |  |
| m[6][8]: | 0.0083(0.0082) | m[6][9]: | 0.0110(0.0108) | m[6][10]: | 0.0125(0.0119) |  |  |
| m[7][8]: | 0.0093(0.0089) | m[7][9]: | 0.0101(0.0094) | m[7][10]: | 0.0093(0.0095) |  |  |
| m[8][8]: | 0.6806(0.0132) | m[8][9]: | 0.0522(0.0273) | m[8][10]: | 0.0139(0.0134) |  |  |
| m[9][8]: | 0.0090(0.0088) | m[9][9]: | 0.6830(0.0145) | m[9][10]: | 0.0086(0.0085) |  |  |
| m[10][8]: | 0.0089(0.0090) | m[10][9]: | 0.0125(0.0118) | m[10][10]: | 0.8662(0.0287) |  |  |

Supplementary 5: Pairwise estimated migration for all sampling sites produced using the program BAYESASS. Note that m[i][j] is the fraction of individuals in population i that are migrants derived from population j (per generation). Next are the mean posterior estimates (and standard errors) for each sample site. Sample sites are coded as follows; 0: Calamity Brook, 1: Nate Brook, 2: Slide Brook, 3: Snyder Brook, 4: Durgin Brook, 5: Vanderwhacker Brook, 6: Unnamed Brook 1, 7: Gulf Brook, 8: Unnamed Brook 2, 9: Platt Brook, 10: Shanty Bottom Brook.
